# Case report: Clinicopathological characteristic of two cases of primary endometrial squamous cell carcinoma and review of the literature

**DOI:** 10.3389/fonc.2024.1415816

**Published:** 2024-08-26

**Authors:** Hui-Bin Zhang, Li-Hua Lin, Qiu-Ping Lin, Yuan-Qing Lin, Dan Luo, Shu-Xia Xu

**Affiliations:** ^1^ Department of Pathology, Fujian Maternity and Child Health Hospital, College of Clinical Medicine for Obstetrics & Gynecology and Pediatrics, Fujian Medical University, Fuzhou, Fujian, China; ^2^ Department of Health Care Section, Fujian Maternity and Child Health Hospital, College of Clinical Medicine for Obstetrics & Gynecology and Pediatrics, Fujian Medical University, Fuzhou, Fujian, China; ^3^ Department of Traditional Chinese Medical Science, Fujian Maternity and Child Health Hospital, College of Clinical Medicine for Obstetrics & Gynecology and Pediatrics, Fujian Medical University, Fuzhou, Fujian, China; ^4^ College of Environment and Public Health, Xiamen Huaxia University, Xiamen, Fujian, China

**Keywords:** primary, endometrial squamous cell carcinoma, pathology, pathogenesis, clinicopathological feature

## Abstract

Primary endometrial squamous cell carcinoma (PESCC) is a rare malignant tumor. To investigate the clinical and pathological features of PESCC, two cases of PESCC in Fujian Maternal and Child Health Hospital were retrospectively studied and the literatures were reviewed. Both of the two cases were menopausal women aged 57–62 years, clinically presenting with “vaginal discharge”. Case 1 was a non-keratinising squamous cell carcinoma with high-risk HPV infection. Tumor infiltrated in deep myometrium with multifocal intravascular thrombus and macro metastases to one pelvic lymph node (1/15) and abdominal aortic lymph node (1/1). Lung metastasis occurred 36 months after the surgery. After surgical resection and without postoperative supplemental therapy, the patient remained tumor-free for 110 months to date. Case 2 had a history of breast cancer for 5 years and long-term intake of aromatase inhibitor drugs without HPV infection. It was a keratinized squamous cell carcinoma. Tumor also infiltrated in deep myometrium with multifocal intravascular thrombus and one pelvic lymph node metastasis (1/18), However, no metastasis was seen elsewhere. To date, the patient survived for 16 months without tumor after surgery. Both of the two cases expressed squamous epithelial markers P40, P63, and CK5/6, but neither expressed PAX8 or PR. Case 1 had diffuse expression of P16, wild-type P53, and ER-negative. Case 2 had negative P16, mutant P53, and focal positive ER. PESCC is often associated with HPV infection and low estrogen levels. However, studies in the literatures have found that P16 expression is not always consistent with HPV infection, indicating that PESCC cannot be easily classified as HPV-associated or non-dependent like cervical cancer. There are two main patterns of P16 and P53 expression, P16-positive/P53 wild-type and P16-negative/P53-mutant, but no positive expression of both has been seen so far. It is worth noting that we reported the second case of PESCC with a history of breast cancer, where the patient had been taking the oral aromatase inhibitor drug (exemestane) for a long period of time to reduce the estrogen level, indicating the low estrogen level may be also a key factor in the pathogenesis of PESCC.

## Introduction

The endometrium is a glandular epithelial tissue and the majority of malignant tumors arising from it are adenocarcinomas. Primary endometrial squamous cell carcinoma (PESCC) is a rare entity, with an incidence accounting for less than 0.5% of all endometrial malignancies (1). The 2020 World Health Organization Classification of Female Genital Tumors provide comprehensive introductions for the most tumor types. However, the description of PESCC is extremely brief, which shows that there is still a lack of knowledge about PESCC.

Here, we present an in-depth research of the clinicopathological features of two cases of PESCC as well as a review of previously published cases. The aim of this paper is to provide practitioners with actionable knowledge to enhance their understanding of this rare malignancy.

## Materials and methods

The database of the Department of Pathology at Fujian Maternal and Child Health Hospital was searched from 2011 to 2023 using the keywords “squamous cell carcinoma”, “endometrium”, and “whole uterus”. Cases were further screened according to the following criteria: 1. The absence of endometrial adenocarcinomas or epithelial neoplasms; 2. The exclusion of primary squamous cell carcinoma of the uterine cervix.

After rigorous screening, only 2 cases met 100% of the diagnostic criteria for PESCC. The clinical data for the two cases of PESCC were complete, including patients’ demographic information, clinical manifestations, surgical methods, and postoperative patient follow-up data (**Table 1**).

**Table 1:** Clinical features of two primary endometrial squamous cell carcinoma.

NO.	Age (years)	Menopausal status	Clinical symptom	Serum SCC value (normal value<1.5ng/mL)	HPV	Treatments	Stage (FIGO)	Follow-up (mo)
1	62	Postmenopausal 10 years	Vaginal drainage for more than 1 year	8.3↑	+16 +42 +83	Thyst/bso/lnd/chemo	IIIC2	110 NED
2	57	Postmenopausal 8 years	Vaginal discharge for 1 month	10.23↑	–	Rhyst/bso/lnd/chemo	IIIC1	16 NED

Thyst, total hysterectomy; Rhyst, radical hysterectomy; bso, bilateral salpingo-ovariectomy; lnd, lymph node dissection; chemo, chemotherapy; NED, no evidence of disease (alive). ↑, higher than 1.5 ng/mL.

Surgical specimens were fixed in 10% neutral buffered formalin solution for an appropriate duration, dehydrated in a graded series of alcohols, and then embedded in paraffin wax. Sections were cut at a thickness of 4 μm and stained with hematoxylin and eosin (HE). Immunohistochemical staining was performed using the EnVision method with a panel of primary antibodies including P16, P53, P40, P63, CK5/6, ER, PR, CKH, CKL, PAX8, and Ki-67. The primary antibodies were purchased from Fuzhou Maixin Company. The antibody kits were also purchased from the same source. All immunohistochemistry was performed with positive controls, and the results were reliable. Some of the important immunohistochemical interpretation criteria were as follows: P53 staining was considered positive/mutant when there was diffuse strong nuclear staining (greater than 80% of the tumor) or all negative or diffuse cytoplasmic with weak nuclear staining; wild-type P53 was considered when there was unequal strong and weak nuclear staining. P16 staining was considered positive when there was diffuse block-like staining of P16 nuclei; negative when there was patchy or no staining. ER/PR tumor cells were interpreted as negative when the nuclear staining was less than 1%, focally positive when the staining was between 1% and 10%, and positive when the staining was greater than 10%.

### Case description

We confirmed the two cases of PESCC with onset age of 57 to 62 years, the mean age was 59.5 years, menopause for 8–10 years. The clinical symptoms were all of vaginal discharge; the vaginal colorultrasound suggested intrauterine effusion; on gross examination, a large mass formed in the uterine body without involving the cervix. Two cases of PESCC had different clinicopathological features (**Table 2**): Case 1 was a non-keratinized type SCC with high-risk HPV infection, whereas case 2 was keratinized type SCC without HPV infection. Both of the two cases were expressed squamous epithelial-derived markers such as P63, P40 and CK5/6. However, the expression of P16 and P53 on the two cases were quite opposite. Although both PESCC cases showed strong invasion features and higher clinical stage, they appeared to have a good prognostic outcome: Case 1 presented pelvic lymph node and abdominal aortic lymph node metastasis at diagnosis, lung metastasis occurred on 36 months after surgery, and then the lung mass was surgically removed in another hospital, follow-up 110 months, and the patient survival without tumor; case 2 also had pelvic lymph node metastasis, and was followed up 16 months, the patient survival without tumor. Pathologic features are summarized in detail on a case-by-case basis as follows:

**Table 2:** Pathological characteristics of two primary endometrial squamous cell carcinoma.

NO.	Pathological type	Depth of Invasion	LVI	Metastasis	IHC					
					P16	P53	ER	PR	Pax8	KI-67
1	Non-keratinized type SCC	>50% Myoinvasion	Present	Pelvic lymph nodes(1/15); abdominal aortic lymph node (1/1); Lung	+	Wild-type	–	–	–	90%+
2	Keratinized type SCC	>50% Myoinvasion	Present	Pelvic lymph nodes(1/18)	–	Mutant	<10%+	–	–	50%+

LVI, lymphovascular invasion; IHC, immunohistochemistry; ER, estrogen receptor; PR, progesterone receptor.

### Case 1

A 62-year-old postmenopausal woman was admitted to our hospital in March 2015 with a complaint of vaginal discharge for over a year. Pelvic ultrasound revealed fluid in the uterine cavity, and the level of squamous carcinoma-associated antigen (SCC) was elevated to 8.3 ng/mL, which is higher than the normal range of 0.00–1.5 ng/mL. HPV DNA testing was positive for high-risk types 16, 42, and 83. Subsequently, a preoperative hysteroscopy with curettage was performed, and no cervical lesions were identified. The endometrial curetting showed squamous cell carcinoma. Then, the patient underwent radical hysterectomy, bilateral salpingo-oophorectomy, and pelvic lymph node dissection. During the surgery, a greyish-yellow nodule of about 0.5 cm was found adjacent to the abdominal aorta, and frozen section examination revealed metastatic squamous cell carcinoma.

**Figure 1:**
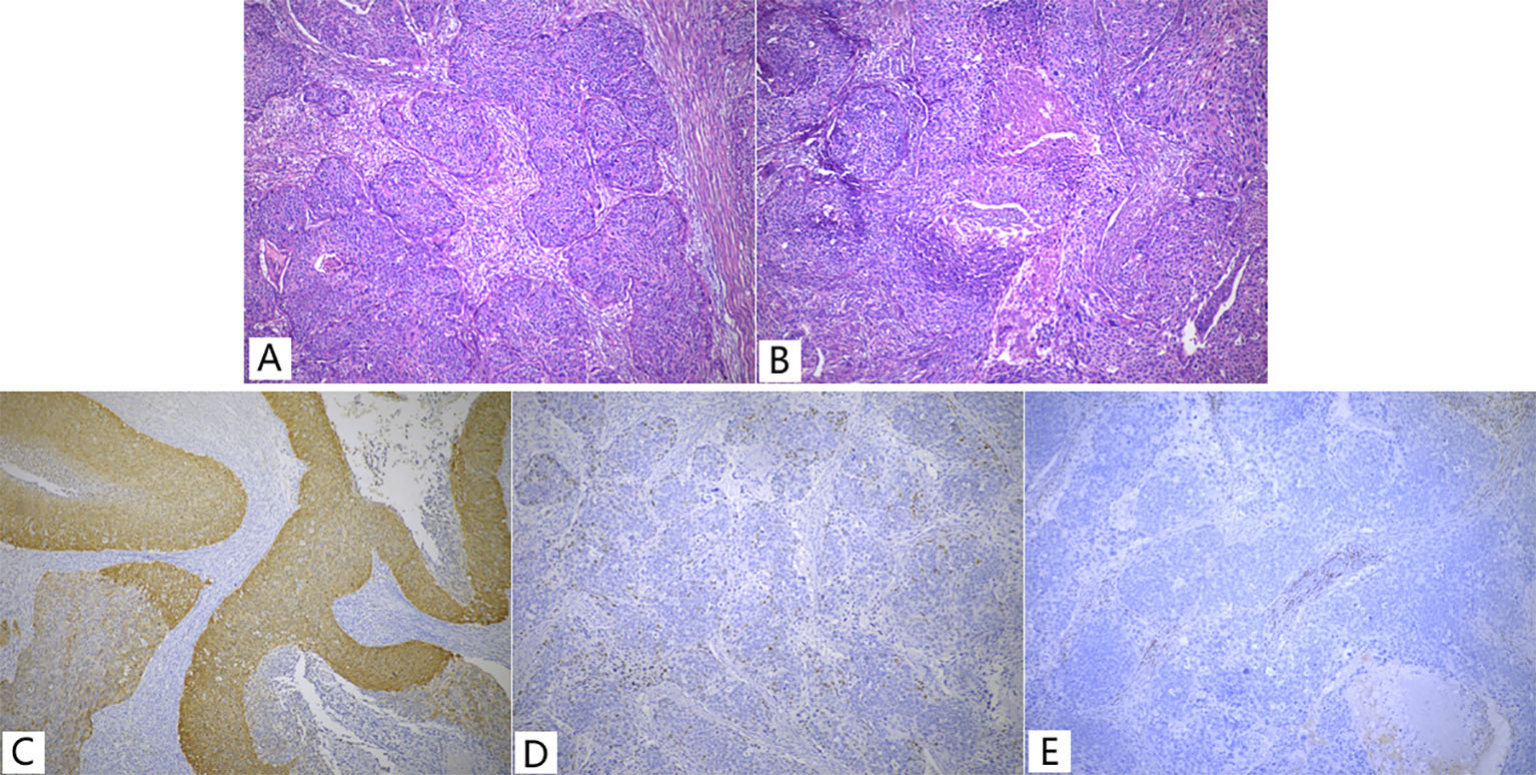
Non-keratinized type SCC **(A, B)** and Immunohistochemistry **(C-E)** of Case 1. **(A)** Showed typical features of non-keratinized squamous cell carcinoma; **(B)** Basal cell carcinoma-like with central necrosis; **(C)** P16 showed diffuse strong positive, a little faded; **(D)** P53, wild-type expression pattern; **(E)** PR, negative,<1%.

On gross examination, the endometrium displayed thickening with an ichthyosis-like appearance and deep invasion (near the plasma layer). Sampling of all cervical tubes did not reveal any intraepithelial lesions. Microscopically, the endometrial mass showed typical non-keratinizing squamous cell carcinoma with tumor cells distributed in nests resembling high-grade intraepithelial lesions involving glands (**Figure 1A**) and some areas appeared basal cell carcinoma-like with central necrosis (**Figure 1B**). Lymphovascular invasion was seen in the mesenchyme. A total of 15 lymph nodes were palpated in the pelvic lymph nodes, and a single metastasis was identified in the left obturator lymph node. Immunohistochemically, the tumor cells were diffusely positive for P63/P40/CK5/6 and P16 (**Figure 1C**), wild-type P53 (**Figure 1D**); negative for ER/PR/Pax8 (**Figure 1E**); and Ki67 index of about 90%. After the surgery, the patient received platinum-based chemotherapy and was followed regularly. At postoperative 36 months, lung metastasis was detected and surgically resected at another hospital, telephone follow-up revealed that the patient had not undergone any additional postoperative treatment. The patient is currently being followed up for 110 months with regular follow-up appointments but has not shown any evidence of disease recurrence to date.

### Case 2

A 57-year-old postmenopausal woman was referred to our hospital in March 2023 for a complaint of vaginal discharge for 1 month. She had a history of oral exemestane therapy for breast cancer for 5 years. Pelvic magnetic resonance imaging (MRI) suggested a mass in the uterine cavity measuring approximately 35×42×63 mm. The serum squamous cell carcinoma-associated antigen (SCC) level was elevated to 10.23ng/mL, while CA125, CA199, and CEA values were within the normal range. HPV E6/E7 mRNA detection was negative for high-risk human papillomavirus.

**Figure 2:**
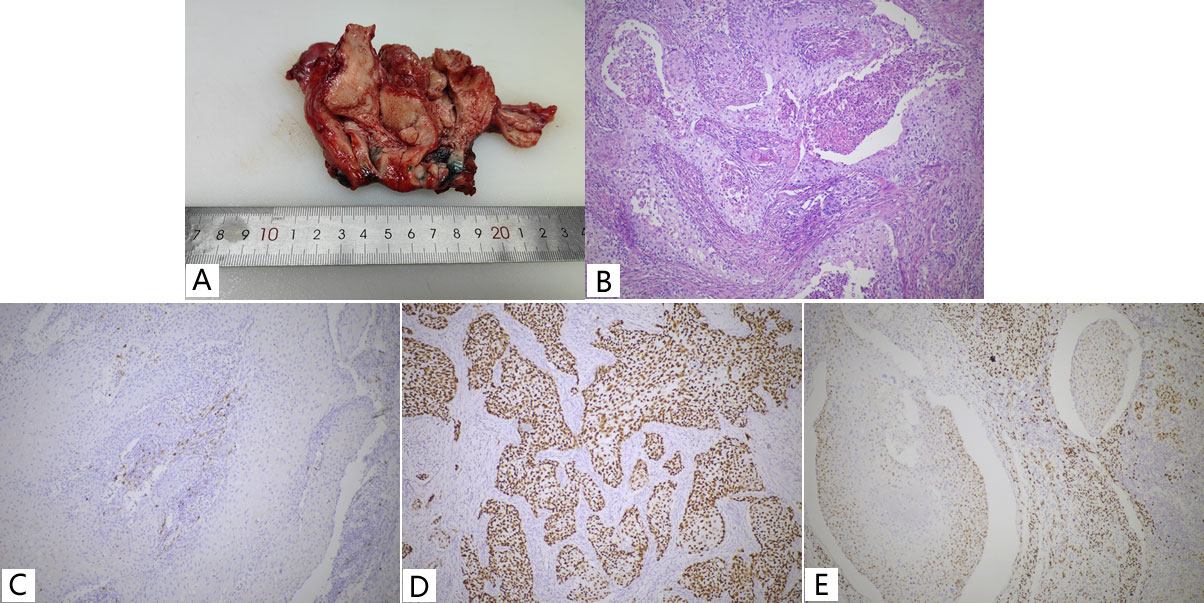
Keratinized type SCC **(A, B)** and Immunohistochemistry **(C-E)** of Case 2. **(A)** A prominent mass in the uterine body; **(B)** Pure keratinized squamous cell carcinoma with large amounts of neutrophils aggregated. **(C)** P16, negative; **(D)** P53, Aberrant/mutation-type expression pattern; **(E)** ER, focally positive, between 1% -10%.

Curettage of the endometrium and endocervix revealed keratinized squamous cells with moderate anisotropy. The patient then underwent radical hysterectomy, bilateral salpingo-oophorectomy, pelvic lymph node, and para-abdominal aortic lymph node dissection. Macroscopically, a bulging mass measuring 40×35×25 mm involved primarily the endometrium(**Figure 2A**), with tumor invasion into the deeper half of the myometrium and lymphovascular space.

Microscopically, the tumor cells showed typical morphological features of keratinized squamous cell carcinoma with abundant eosinophilic cytoplasm and keratin pearls (**Figure 2B**), and the cervix was free of disease. A total of 18 lymph nodes were palpated in the pelvic lymph nodes and a single metastasis was identified. Immunohistochemical analysis demonstrated strong positive staining for P63/P40/CK5/6, negative staining for P16/PR, focal positive staining for ER, nearly 100% strong positive staining for P53, and Ki-67 index of about 50% (**Figures 2C-E**).

The patient received chemotherapy with paclitaxel and carboplatin after surgery and has been free of disease for 16 months.

## Discussion

Most malignancies occurring in the endometrium are adenocarcinomas, and PESCC is an extremely rare malignancy. Due to the diagnostic criteria for PESCC is stringent and it belongs to an exclusion diagnosis, from 2011 year to 2023 year (as of August 31) in the Department of Pathology, Fujian Maternal and Child Health Hospital, a total number of 1999 of cases of endometrial cancer were reported, including only 2 cases of PESCC were revealed. It accounted for about one in thousand.

The earliest and traditional diagnostic criterion, still in use today, was proposed by Fluhmann (2) in 1928 and requires fulfillment of all three of the following criteria: 1) the absence of endometrial adenocarcinomas or epithelial neoplasms; 2) the absence of endometrial squamous cell carcinoma in association with the squamous epithelium of the uterine cervix; and 3) the absence of primary squamous cell carcinoma of the uterine cervix. However, some pathologists recognize that the diagnostic criteria for PESCC should not be restricted to excluding involvement of the cervical canal because tumors are always invasive. Mark et al. (3) reported four cases of endometrial squamous cell carcinoma involving the cervical canal that were confirmed to be PESCC by modern adjuvant techniques such as immunohistochemistry, HPV *in-situ* hybridization, and molecular testing. This suggests that the diagnostic criteria for PESCC can be appropriately relaxed to allow for the presence of involvement of the cervical canal, but this also increases the difficulty of diagnosis because there are not rare cases of squamous cell carcinoma of the cervix involving the endometrium. Therefore, to achieve a more accurate diagnosis, it is necessary to fully understand the clinicopathological features of PESCC.

PESCC patients are mostly seen in perimenopausal or postmenopausal women (4), with an average age of onset of approximately 67 years, and very rarely observed in women of childbearing age (5, 6). The clinical manifestations of PESCC are similar to those of endometrioid carcinoma, with the most common presentation being vaginal bleeding, followed by uterine pus and vaginal discharge, and some patients are found to have uterine fibroids occupying the uterus on further physical examination (7–10). The pathogenesis of PESCC is still unclear, but it is generally believed that the endometrium is stimulated by various factors leading to the squamous development of endometrial precursor cells, which ultimately leads to carcinoma. These causative factors include uterine pus accumulation, uterine fibroids, chronic endometritis, and pelvic radiation, etc. (4–7). A small number of cases of PESCC have been reported to occur as a secondary effect of hormonal therapy or radiotherapy treatment for breast, colorectal and ovarian cancers (8–11). One of the breast cancer patients was on long-term tamoxifen use, which is similar to our reported case 2 patient with a history of breast cancer and long-term use of an estrogen antagonist drug (exemestane). This indicates that low estrogen levels may also be a contributing factor in the development of PESCC.

It is well established that the majority of cervical squamous cell carcinomas are associated with HPV infection. However, the relationship between HPV infection and PESCC remains a topic of debate. Currently, the World Health Organization (WHO) recognizes an association between HPV infection and PESCC. Kataoka et al. (12) were the first to demonstrate that one case of PESCC was positive for HPV type 31 by PCR, and they proposed that HPV infection might be related to the development of PESCC. Subsequently, Darré et al. (5) and Zhang et al. (13) also reported two cases of HPV-associated PESCC. However, many cases of PESCC to date have not been associated with HPV infection, and most of these cases have undergone immunohistochemistry or molecular testing. Most cases of PESCC were found to have abnormal expression or mutation of the oncogene P53 by immunohistochemistry or molecular testing, suggesting that mutation of the oncogene P53 is associated with the development of PESCC (3, 6, 14).

The microscopic appearance and morphological characteristics of PESCC are generally like those of squamous cell carcinoma of the uterine cervix, being predominantly keratinized or non-keratinized types with the two types being able to coexist, and a small proportion being papillomavillous or uroepithelial cells (15, 16).

Immunohistochemistry demonstrates that PESCC expresses squamous differentiation markers such as P40, P63, and CK5/6, and generally lacks Müllerian-derived markers such as PAX8, ER, PR, but ER may be focally expressed in a small number of cases (3), and only one case of PAX8 positivity has been reported so far (17). In addition, there are two patterns of P16 and P53 expression in PESCC: one being positive for P16 with wild-type P53 expression and the other one is negative for P16 with abnormal expression of P53. A small proportion of PESCC are negative for both P16 and P53 markers, indicating the activation of other pathogenic molecular pathways involved in the development of PESCC. Reviewing the literature, there are no cases of positive expression of both P16 and P53 markers, suggestive of a potential mutual exclusivity in the expression of the two in PESCC; however, more data are required to verify this phenomenon, which could aid in distinguishing plasmacytoid carcinomas with extensive squamous differentiation. Additionally, Horn et al. (18) only detected HPV in one out of four cases of P16-positive PESCC, indicating that p16-positive PESCC may not always be HPV-associated and that classification based on HPV status may not be appropriate for PESCC. Molecular alterations in PESCC involve TP53, CDKN2A, pRB-CyclinD1-CDK4/6-p16, PTENT, and PIK3CA (3, 19), and only one case of PESCC has been molecularly typed as MSI-H (20).

PESCC requires differential diagnosis from multiple tumors. The first differential diagnosis is endometrial adenocarcinoma with extensive squamous metaplasia or adenosquamous carcinoma, which is the most common malignancy of the endometrium, often associated with squamous metaplasia and mucinous metaplasia. When endometrial adenocarcinoma is associated with extensive squamous metaplasia, the squamous component may completely cover the glandular component, leading to misdiagnosis as squamous cell carcinoma. However, immunohistochemistry cannot help distinguish between the two, so comprehensive sampling of the endometrium is necessary to determine the presence of endometrial glandular epithelial lesions in the diagnosis of primary squamous cell carcinoma of the endometrium. Even a small amount of glandular epithelial lesion suggests that the tumor may be more common endometrial cancer with squamous metaplasia rather than squamous cell carcinoma of the endometrium. The second differential diagnosis is cervical squamous cell carcinoma involving the endometrium. According to Fluhmann’s (2) criteria, when squamous cell carcinoma involves both the cervix and endometrium, it is classified as cervical with endometrial involvement. However, occasionally, PESCC may involve the cervix. All reported cases of this type of PESCC are non-HPV-related and often associated with abnormal P53 expression and negative P16 staining (3), unlike primary cervical squamous cell carcinoma, which is mostly HPV-related, P16-positive, and shows wild-type P53 expression. The final differential diagnosis is from squamous differentiation in papillary serous carcinoma: squamous differentiation is rare in papillary serous carcinoma. Santoro et al. (21) reported a case of endometrial papillary serous carcinoma with extensive squamous differentiation mimicking primary endometrial squamous cell carcinoma. This case presented with features of complete squamous differentiation, expressing squamous markers such as P40, P63, and CK5/6, but not Müllerian-derived markers such as PAX8, ER, PR, etc. P53 was aberrantly expressed and P16 was limited to basal expression. The diagnosis was consistent with primary squamous cell carcinoma, but morphological and immunohistochemical features of papillary serous carcinoma were seen in the sentinel lymph node. For this rare and atypical case, only comprehensive sampling can reduce unnecessary misdiagnosis.

Due to the rarity of PESCC cases, there is no consensus on the treatment of this disease. Early on, Goodman et al. (4) proposed total hysterectomy with double adnexectomy as the optimal therapy for PESCC. Subsequently, Kennedy et al. (22) reported the first case of PESCC treated with postoperative adjuvant chemotherapy using cisplatin, leading to the development of a treatment pattern based on surgery with postoperative combined radiotherapy and/or chemotherapy. However, there is no recommendation for the optimal combination of modalities. Song et al. (20) analyzed the survival data of 31 cases of PESCC and recommended that surgery remain the main treatment modality for PESCC, with lymph node clearance being advocated. For stage I patients, appropriate radiotherapy could be given after surgery, while for stage III and IV patients, combined radiotherapy and chemotherapy should be given. Molecular typing is recommended because some patients may benefit from immunotherapy such as PD-L1.

Some studies have indicated that PESCC has a poorer prognosis than endometrioid carcinoma (4). Furthermore, the prognosis correlates with pathological staging. A five-year survival analysis conducted by Varras and Kioses revealed that the prognosis for patients diagnosed with early-stage PESCC was significantly more favorable than that of patients diagnosed with late-stage disease (23). The most common causes of death were tumor recurrence and metastasis, with pelvic metastasis being the most prevalent, followed by lung, brain and bone metastases (4, 23). Nevertheless, data from Dalrymple et al. (9) on four patients with PESCC followed up for up to 10 years indicated that the prognosis of even advanced PESCC with distant metastases was not as poor as previously thought. Our case 1, which involved metastases to the lungs, was monitored for over nine years with a favorable prognosis. However, due to the limited number of cases, this does not provide a representative overview of the overall prognosis. PESCC can be comorbid with other types of tumors, as evidenced by a case of PESCC combined with early tubal adenocarcinoma reported by Caulkins et al. (24) and a case of PESCC combined with neuroendocrine tumors reported by Varras and Kioses (23). Similarly, the diagnosis of PESCC necessitates the exclusion of metastasis of SCC from other sites. A case has been reported of vulvar cancer metastasizing to the endometrium of the uterus without involvement of the cervix (25). The presence of these combined higher-grade tumors may contribute to the poor prognosis observed in some cases of PESCC.

## Conclusion

The clinical features of the two cases of PESCC we reported here were consistent with those reported in the literature. One case being accompanied by high-risk HPV infection, suggesting that there is a correlation between the occurrence of PESCC and HPV infection. However, a review of the literature showed that the expression of P16 did not coincide with HPV infection, indicating that PESCC is not suitable for simple classification based on HPV-associated or non-dependent cervical cancer classification. PESCC expressed the squamous epithelial markers such as P40, P63 and CK5/6, most of which did not express the mullerian origin markers PAX 8, PR and ER. There were mainly two patterns of P16 and P53 expression, i.e., P16-positive/P53 wild-type and P16-negative/P53-mutant. Only a small number of them were negative for both, but none of them was found to be positive, which suggested that P16 and P53 may be mutually exclusive and more data were needed to verify this. In addition, it is worth mentioning that we reported the second case of PESCC with a history of breast cancer who had been taking the aromatase inhibitor drug “exemestane” for a long time to reduce the estrogen level, indicating a low estrogen level may be also a risk factor for the pathogenesis of PESCC.

## Data Availability

The original contributions presented in the study are included in the article/supplementary material. Further inquiries can be directed to the corresponding authors.
